# Supplementary material for: Whole-genome sequencing of Burkholderia glumae strains from Thailand reveals potential horizontal gene transfer with Burkholderia pseudomallei
Source: PLoS One. 2025 Dec 31;20(12):e0340071. doi: 10.1371/journal.pone.0340071 (PMC12755741; doi:10.1371/journal.pone.0340071)
Supplement: Attachment — Submitted filename: Response to Reviewers.pdf [file pone.0340071.s004.pdf]

Response to the reviewers' comments

Editor Comments:

The study provides valuable insights into the genomic heterogeneity and interspecies gene transfer between *B. glumae* and *B. pseudomallei.* Below, additional suggestion:

In the Introduction, please clarify the ecological overlap between *B. glumae* and *B. pseudomalleii,* briefly stating how this shared niche could facilitate genetic exchange.

We thank the editor for this helpful suggestion. We have revised the Introduction to more clearly describe the ecological overlap between *B. glumae* and *B. pseudomallei*, and to explain how this shared habitat may promote opportunities for genetic exchange.

The original text:

"Given that *B. glumae* and *B. pseudomallei* share the environmental niche of rice fields, it is highly possible that they coexist in Thai rice fields and interact directly. However, few studies have investigated the specific interactions between *B. glumae* and *B. pseudomallei*."

Has been revised to:

"Given that *B. glumae* and *B. pseudomallei* share the environmental niche of rice fields, it is highly likely that they coexist in Thai rice paddies and interact directly. In addition, the prevalence of both species increases with higher humidity and rainfall [2,6]. As a result, the population densities of both organisms are likely to peak simultaneously during the high-precipitation rice-growing season. The coincident population dynamics of *B. glumae* and *B. pseudomallei* may intensify their niche sharing, resulting in frequent cell-to-cell contact within the highly active and dense microbial communities of waterlogged rice-field soils. These high-density and environmentally stressed conditions provide an optimal setting for genetic exchange, thereby increasing the likelihood of horizontal gene transfer (HGT) mediated by mobile genetic elements or bacteriophages [7,8]."

Highlight novelty and research gap, by explicitly state what remains unknown (e.g., genomic evidence of interspecies HGT in B. glumae from Thailand).

We thank the editor for this constructive feedback. We have revised the Introduction to more clearly emphasize the novelty of our study and to explicitly identify the existing research gap. In particular, we now state that genomic evidence of interspecies HGT in *B. glumae* isolates from Thailand has not been previously investigated.

The original text:

"Together with the knowledge that *B. glumae* and *B. pseudomallei* share an environmental habitat in Thailand, the documented capacity for HGT within the *Burkholderia* genus indicates that it is critical to determine whether these species exchange genes. Such gene exchange may have implications for the evolution of virulence in *B. glumae* and may alter the pathogenic landscape in agricultural and public health contexts."

Has been revised to:

"The coexistence of *B. glumae* and *B. pseudomallei* in Thai environments, coupled with extensive evidence of horizontal gene transfer within *Burkholderia*, makes it critical to determine whether these two species exchange genes in nature. However, no study to date has examined genomic evidence of interspecies HGT in *B. glumae* isolates from Thailand. Addressing this gap is essential, as such gene exchange may influence the evolution of virulence, environmental adaptation, and the broader pathogenic landscape with implications for both agriculture and public health."

Emphasize rationale and objectives, explaining why whole-genome sequencing and comparative genomics are the best approaches for addressing the study question.

Thank you for this very helpful suggestion. We have revised the Introduction to clearly describe the rationale for using whole-genome sequencing (WGS) and comparative genomics, and to explain why these methods are appropriate for addressing our study objectives.

The original text:

"In this study, we performed whole-genome sequencing (WGS) and a comparative genomic analysis of 16 *B. glumae* isolates from Thailand. Our objective was to evaluate the potential for HGT events between *B. glumae* and other *Burkholderia* species, particularly *B. pseudomallei*. Our findings provide new insights into the evolutionary dynamics of *B. glumae* and contribute to a better understanding of its genomic relationships with clinically important *Burkholderia* species."

Has been revised to:

"In this study, we performed whole-genome sequencing (WGS) and a comparative genomic analysis of 16 *B. glumae* isolates from Thailand to investigate evidence of HGT events between *B. glumae* and other *Burkholderia* species, particularly *B. pseudomallei*. WGS combined with comparative genomics allows high-resolution detection of mobile genetic elements, horizontally acquired regions, and evolutionary relationships across species. This approach is therefore well suited for identifying HGT events and elucidating genome evolution in *Burkholderia* [14,15]. Our findings provide new insights into the evolutionary dynamics of *B. glumae* and contribute to a better understanding of its genomic relationships with clinically important *Burkholderia* species."

Strengthen the opening paragraph of the Discussion, expanding slightly on how your findings relate to existing knowledge on *B. glumae* genomics and HGT in *Burkholderia* species.

We thank the editor for this valuable comment. We have revised the opening paragraph of the Discussion to better contextualize our findings within the existing literature on *B. glumae* genomics and HGT in *Burkholderia* species. The revised text now more clearly highlights what is known, what remains insufficiently studied, and how our findings advance current understanding.

The original text:

"The findings of this study provide insights into the genomic composition of *B. glumae* populations in Thailand and support the hypothesis that HGT plays an important role in shaping the genomic plasticity of this pathogen. Furthermore, they indicate that interspecies HGT may occur between *B. glumae* and *B. pseudomallei*."

Has been revised to:

"The findings of this study provide new insights into the genomic composition of *B. glumae* populations in Thailand and support the hypothesis that HGT plays an important role in shaping the genomic plasticity of this pathogen. Previous genomic studies of *B. glumae* have largely focused on pathogenicity factors, host interactions, and population diversity, with limited investigation into HGT. Although previous studies have reported HGT events from distinct soil bacterial taxa such as *Pseudomonas* and *Streptomyces* [14,15], the investigation of HGT between *B. glumae* and other *Burkholderia* species remains limited, particularly in environments where *B. glumae* shares the same habitat with human-pathogenic *Burkholderia*, as observed in Thailand."

Discuss possible mechanisms or ecological contexts facilitating HGT (e.g., soil or plant-associated microbial communities in rice fields).

Thank you for this insightful comment. In response, we have substantially expanded the Discussion to describe the ecological and mechanistic factors that may facilitate horizontal gene transfer (HGT) between *B. glumae* and *B. pseudomallei* in rice-field environments.

The original text:

"Thus, in areas of Thailand where rice fields harbor both *B. glumae* and *B. pseudomallei*, horizontal transfer of genes between these species is possible. Such HGT may be driven by selective pressures, such as environmental stress, phage predation, and microbial competition [55]."

Has been revised to:

"This geographic co-occurrence provides direct ecological opportunity for interspecies contact. In addition, both species exhibit higher prevalence during periods of increased rainfall and humidity [2,6], which lead to simultaneous population peaks during the rice-growing season. These seasonal conditions create dense, metabolically active microbial communities in waterlogged paddy soils, enhancing the frequency of cell-to-cell contact, and the mobilization of plasmids and prophages [7]. Phage activity is also likely to be intensified under these environmentally stressed, nutrient-fluctuating conditions. Phages are known to adapt to such environments by modulating infection strategies, expanding host ranges, and expressing auxiliary metabolic genes that enhance microbial stress tolerance [8]. Moreover, phage-driven processes such as phage predation and the viral shunt can increase bacterial DNA release and uptake, activate mobile genetic elements, and thereby enhance the potential for phage-mediated horizontal gene transfer [8,55]. In addition, bacterial antiviral systems, which balance the costs of cell lysis with the benefits of acquiring new genes, may further modulate the likelihood of successful gene exchange [8]. Consequently, in rice-field settings where *B. glumae* and *B. pseudomallei* overlap, these combined ecological and mechanistic drivers likely facilitate interspecies HGT, offering a plausible explanation for the gene transfer events observed in this study."

Mention any study constraints such as small sample size, limited geographic or temporal coverage, or lack of expression data for transferred genes.

Thank you for this helpful suggestion. We have added a limitations statement prior to the section on future directions in the Discussion.

The added text reads:

"Several limitations should be acknowledged. Although the 16 isolates analyzed in this study were collected from multiple geographic regions and different years, the overall sample size remains small, which may limit the generalizability of the observed population structure and HGT patterns. In addition, our conclusions are based solely on genomic data, without functional validation of the putative horizontally transferred genes."

Revise the final paragraph of the Discussion to strengthen the conclusion and emphasize the broader implication of the study for both plant and human health.

Thank you for this valuable suggestion. We have revised the final paragraph of the Discussion to strengthen the overall conclusion and emphasize the broader implications of our findings for both plant and human health.

The original text:

"Thus, the acquisition of virulence genes by *B. glumae* from human pathogenic *Burkholderia* species, particularly *B. pseudomallei*, may increase *B. glumae*'s pathogenicity and host range."

Has been revised to:

"Our findings suggest that similar gene flow may be occurring between *B. pseudomallei* and *B. glumae*. The acquisition of virulence-associated genes by *B. glumae* from human-pathogenic *Burkholderia* could have important consequences, potentially enhancing its pathogenicity, expanding its host range, and altering its ecological behavior. These observations underscore the need for continued genomic surveillance using One Health perspective to monitor the emergence of traits that may impact both agricultural productivity and public health."

Review Comments to the Author

Reviewer 1:

As stated in the text, strains 60BGCRMSO1-5, 60BGCRMSO3-5, 60BGCRMSO3-9, 60BGCRMSO3-11, 60BGCRWC8-5, and 60BGCRPA10-1 (reported in Table 1) were previously subjected to whole genome sequencing (WGS) in another manuscript (https://doi.org/10.3390/pathogens11060676). Therefore, I suggest distinguishing between re-sequenced and de novo sequenced strains in Table 1. Were the results obtained by re-sequencing consistent with those previously obtained? Furthermore, I recommend adding columns for the geographic coordinates of the isolation locations and the rice cultivars to Table 1.

Thank you for this helpful comment. We have updated Table 1 to clearly distinguish between de novo–sequenced strains and strains that were previously sequenced and re-sequenced in this study. An additional column titled "Sequencing status" has been added to indicate whether each isolate underwent de novo sequencing or re-sequencing. We also added a column for rice cultivar, as suggested. However, geographic coordinates were not available for these isolates; therefore, we retained the province of origin, which represents the most precise location information available for this dataset.

To reflect these changes, the Table 1 title has also been updated to:

"Table 1. Isolation information and sequencing status of *B. glumae* strains analyzed in this study."

For the strains that were re-sequenced, the assemblies generated in our study provide substantially better completeness compared with the assemblies in the previous report (https://doi.org/10.3390/pathogens11060676). The previous study used only short-read sequencing, which resulted in highly fragmented assemblies (>100 contigs) and limited the downstream analyses such as determining gene localization, reconstructing plasmids, and mapping transposable elements. In this study, we employed a hybrid sequencing approach that combined short- and long-read data to obtain complete genome assemblies, including closed chromosomes and plasmids. These high-quality assemblies made it possible to accurately assess genomic localization, plasmid composition, and transposable elements.

L96. Why were B. glumae isolates collected from rice fields grown at 37°C rather than at a lower temperature for DNA extraction?

Thank you for pointing out this issue. The temperature reported in the manuscript was incorrect. The isolates were actually grown at 35°C, not 37°C. We have corrected this in the revised manuscript. The use of 35°C follows the optimum temperature for *B. glumae* from previous report (30-35°C) (doi: 10.1111/j.1364-3703.2010.00676.x). No steps in our workflow involved incubation at 37°C.

We selected 35°C over 30°C to optimize DNA purity for Nanopore sequencing. DNA extracted from cultures grown at 30°C consistently showed low A260/230 ratios, likely due to metabolite contamination that was not efficiently removed by the extraction kit. Such metabolites can adversely affect long-read sequencing performance. Cultivating the isolates at 35°C yielded DNA of substantially higher purity suitable for high-quality Nanopore sequencing.

Fig.2 should be modified to improve clarity. The province at the top of the heatmap is not very clear.

Thank you for this helpful comment regarding the clarity of Figure 2. To address this, we have updated the figure by modifying the color palette used for province annotations to enhance visual distinction and readability. In addition, we increased the size of the annotation boxes to ensure that the labels are more easily interpretable. The revised Figure 2 now provides clearer visualization of province-level metadata.

Please use the same abbreviation for Average Nucleotide Identity (ANI) in the text and in Table S1. I suggest writing the full name of ANI in the legend of the table.

We appreciate the reviewer's careful review of the supplementary table and for pointing out this correction. In response, we have corrected the typographical error in Table S1 by replacing AIN with ANI, and we have added the full term "Average Nucleotide Identity (ANI)" to the legend of Table S1 for clarity.

L346. B. pseudomallei should be written in italics.

We appreciate the reviewer for pointing out this formatting detail. The genus–species name *B. pseudomallei* has now been corrected to italics at Line 346.

Reviewer 2:

Line 26 – The term genomic plasticity is not sufficiently defined. Please clarify what is meant in this context (e.g., genome rearrangements, mobile elements, recombination, or overall genomic variability).

We thank the reviewer for this helpful suggestion. The sentence has been revised to clarify what is meant by "genomic plasticity." Specifically, we now define genomic plasticity as including frequent genome rearrangements, variability in mobile genetic elements, and recombination events that facilitate horizontal gene transfer.

The original text:

"Given the high genomic plasticity and frequent horizontal gene transfer observed in *Burkholderia* species, there are concerns about the emergence of novel traits that may affect both plant and human health."

Has been revised to:

"Given the high genomic plasticity of *Burkholderia* species, including frequent genome rearrangements, variability in mobile genetic elements, and recombination events that facilitate horizontal gene transfer, there are concerns about the emergence of novel traits that may affect both plant and human health."

In addition, we have expanded the explanation of genomic plasticity in the Introduction to ensure that the concept is clearly defined earlier in the manuscript.

The original text:

"Members of the *Burkholderia* genus exhibit remarkable genomic plasticity, which is facilitated by frequent HGT events within and between Burkholderia species [6,7]."

Has been revised to:

"Members of the *Burkholderia* genus exhibit remarkable genomic plasticity driven by genome rearrangements, recombination events, and the acquisition of diverse mobile genetic elements such as plasmids, integrative elements, and prophages, which facilitate HGT within and between *Burkholderia* species [6,7].

These revisions provide a clearer and more comprehensive definition of genomic plasticity in the context of *Burkholderia* biology.

Line 83 – If the putative mobile genetic elements were transferred in the past, the past tense should be used.

We appreciate the reviewer's comment. To reflect that the horizontal gene transfer (HGT) events occurred historically rather than prospectively, we have revised the sentence to avoid implying future or potential transfer.

The original text:

"In this study, we performed whole-genome sequencing (WGS) and a comparative genomic analysis of 16 *B. glumae* isolates from Thailand. Our objective was to evaluate the potential for HGT events between *B. glumae* and other *Burkholderia* species, particularly *B. pseudomallei*."

Has been revised to:

"In this study, we performed whole-genome sequencing (WGS) and a comparative genomic analysis of 16 *B. glumae* isolates from Thailand to investigate evidence of HGT events between *B. glumae* and other *Burkholderia* species, particularly *B. pseudomallei*."

Line 99 – The genomic DNA extraction method (scraping colonies from agar plates) is suboptimal, often resulting in lower-quality DNA. While this may have sufficed for sequencing, it is not ideal. Please provide justification or references supporting this method.

Thank you for this comment. We have added a reference supporting the use of plate-grown bacterial cells for high-quality long-read DNA extraction.

Comparative evaluation of commercial DNA isolation approaches for nanopore-only bacterial genome assembly and plasmid recovery. Sci Rep. 2024;14: 27672. doi:10.1038/s41598-024-78066-2

The authors of the referenced study were also collaborators in our project. They observed that liquid cultures grown under shaking conditions often produced DNA with lower structural robustness and increased fragmentation after extraction. In contrast, cells harvested from solid media yielded DNA with substantially greater integrity and longer fragment lengths, which are more suitable for long-read Nanopore sequencing. Therefore, the DNA extraction approach used in our study aligns with current best practices for maximizing long-read sequencing quality.

Line 147 – Consider citing relevant literature describing the specific Burkholderia strains mentioned, to provide more context and support for the statements made.

Thank you for this suggestion. The relevant references describing the *Burkholderia* strains mentioned at Line 147 are already included in the manuscript immediately following the strain names. We have rechecked the text to ensure that the citations are clearly visible and correctly formatted.

Line 162 – The statement that "the strains were distributed across clades" is somewhat tautological; any phylogenetic analysis will, by definition, place strains across clades. Consider rephrasing to highlight a meaningful observation (e.g., clustering pattern, host-specific associations, or geographic structure).

Thank you for this helpful comment. We agree that the original phrasing did not adequately convey the biological relevance of the observation. As mentioned in the introduction, *B. glumae* is considered an emerging rice pathogen in Thailand and is thought to have been introduced from other countries before spreading locally. Under this scenario, limited genetic diversity would be expected. However, our analysis revealed that the 16 Thai strains were separated into several distinct, well-supported clades rather than clustering together, suggesting that *B. glumae* was introduced into Thailand multiple times. To clarify this point, we have revised the sentence accordingly.

The original text:

"The genomes of the 16 Thai *B. glumae* strains were analyzed alongside those of strains from other countries to investigate their phylogenetic relationships. A maximum-likelihood tree generated from core-genome SNP data revealed that the Thai strains exhibited high genetic diversity (Fig 1). The 16 strains were distributed across multiple clades."

Has been revised to:

"The genomes of the 16 Thai *B. glumae* strains were analyzed alongside publicly available genomes from multiple countries to contextualize the evolutionary relationships of Thai strains within the global population structure. A maximum-likelihood tree generated from core-genome SNP data revealed that the Thai strains were separated into multiple well-supported clades rather than clustering together (Fig 1), indicating the presence of distinct *B. glumae* populations."

Line 164 – The statement that strains had minimal SNP divergence seems inconsistent with the earlier claim of high genetic diversity. Please clarify or reconcile these points.

Thank you for this insightful comment. The minimal SNP divergence applies only to the monophyletic subgroup that appears to represent a globally circulating epidemic strain, whereas the overall strain collection displays broader phylogenetic diversity. We agree that mentioning minimal SNP divergence immediately after stating that the Thai strains exhibited high genetic diversity could appear inconsistent. To address this, we have revised the paragraph to remove the general statement of “high genetic diversity” and instead clearly describe the distribution of the strains across multiple phylogenetic clades, indicating the presence of distinct *B. glumae* populations. We then separately describe the monophyletic clade that shows minimal SNP divergence. This restructuring clarifies that the low divergence applies only to that specific clade and avoids any perceived inconsistency. The revised text is included above in our response to the previous comment.

Line 166 – Past tense (“had spread”) would be more appropriate if referring to a historical dissemination event.

Thank you for this helpful comment. We agree that past tense is more appropriate when describing a historical dissemination event. Accordingly, we have revised the sentence at Line 166 to use the past tense.

The original text:

“This suggests the presence of a clonal lineage spread across geographically distant regions.”

Has been revised to:

“This suggests the presence of a clonal lineage that had spread across geographically distant regions.”

Line 193 – Please clarify that this represents a negative finding (absence of prediction) rather than evidence for the absence of the feature, or provide data supporting the conclusion.

Thank you for this critical comment. We agree that the original wording may have implied definitive biological absence rather than a lack of predicted plasmid. To avoid overinterpretation, we have revised the sentence to clarify that this is a negative prediction based on the plasmid identification tools used. The updated text now states that no plasmids were predicted to originate from other bacterial taxa.

The original text:

"Importantly, all detected plasmids were species specific, with no evidence of plasmids originating from other bacterial taxa."

Has been revised to:

"Importantly, all detected plasmids were predicted to be species specific, and no plasmids were predicted to originate from other bacterial taxa based on the tools used."

Line 282 – Use the past tense ("had occurred") for events of horizontal gene transfer. Moreover, because genomic plasticity is not clearly defined, it is difficult to interpret the meaning of this statement.

Thank you for this comment. We agree that past tense is appropriate when referring to historical HGT events.

The original text:

"Furthermore, they indicate that interspecies HGT may occur between *B. glumae* and *B. pseudomallei*."

Has been revised to:

"Furthermore, they indicate that interspecies HGT had occurred between *B. glumae* and *B. pseudomallei*."

Additionally, we clarified the meaning of "genomic plasticity" earlier in the manuscript, as mentioned in the previous comment, to specify that it encompasses genome rearrangements, diversity of mobile genetic elements, and recombination-driven horizontal gene acquisition.

Line 284 – The phrasing about high genetic diversity could be reconsidered. High genomic diversity is expected among environmental Burkholderia isolates, so please specify in what sense this observation is noteworthy.

Thank you for this constructive comment. We agree that high genomic diversity is generally expected among environmental *Burkholderia* species. However, *B. glumae* in Thailand has been considered a recently emerging and likely imported rice pathogen, for which a limited diversity would be expected. We have therefore revised the text to

clarify why the observed diversity is noteworthy in this specific epidemiological context. The revised text explains that, contrary to expectations for a recently introduced pathogen, the Thai isolates show considerable genomic heterogeneity, supporting a polyclonal population structure rather than expansion from a single introduction.

The original text:

"The SNP-based phylogenomic analysis, which included global isolates, revealed high genetic diversity among the 16 Thai B. glumae strains. These results indicate that there is a polyclonal population structure and genomic heterogeneity among the B. glumae strains in Thailand."

Has been revised to:

"The SNP-based phylogenomic analysis, which included global isolates, revealed considerable genetic diversity among the 16 Thai *B. glumae* strains. Because *B. glumae* in Thailand is thought to be a recently emerging and imported pathogen [4], limited diversity would be expected. The observed genomic heterogeneity among the Thai strains therefore supports a polyclonal population structure rather than expansion from a single introduction."

Line 288 – Please clarify whether the strains were isolated in Thailand or processed there by a research group; the current wording is ambiguous.

Thank you for pointing out this ambiguity. To clarify, the Thai strains used in this study were isolated in Thailand, whereas the strains from Korea, Japan, Vietnam, and the USA were retrieved from publicly available genome databases and not processed in Thailand. We have revised the sentence to clearly distinguish between isolates collected in Thailand and reference strains obtained from other countries.

The original text:

"In addition, the identification of a distinct monophyletic clade comprising five Thai strains and strains from Korea, Japan, Vietnam, and the USA suggests the presence of a globally disseminated clonal lineage."

Has been revised to:

"In addition, the identification of a distinct monophyletic clade comprising five Thai strains and database-retrieved strains from Korea, Japan, Vietnam, and the USA suggests the presence of a globally disseminated clonal lineage."

Line 296 – The plasmids are only predicted to exist; please make this clear in the text.

Thank you for this helpful comment. We agree that the original phrasing may have implied experimentally confirmed plasmids rather than computational predictions. To avoid overinterpretation, we have revised the text to explicitly state that plasmids were predicted based on the plasmid identification tools.

The original text:

"All 16 Thai *B. glumae* strains contained at least one plasmid, which is consistent with the findings of previous genomic studies showing that plasmids are commonly found in *B. glumae* [31,32]. Notably, all the identified plasmids were specific to *B. glumae*, and no plasmids originating from other bacterial species were detected, indicating species-specific plasmid content among the Thai isolates."

Has been revised to:

"All 16 Thai *B. glumae* strains were predicted to contain at least one plasmid, which is consistent with the findings of previous genomic studies showing that plasmids are commonly found in *B. glumae* [31,32]. Notably, all predicted plasmids were specific to *B. glumae*, and no plasmids were predicted to be derived from other bacterial taxa, indicating species-specific plasmid content among the Thai isolates."

Line 308 – The phrase "facilitate HGT" suggests a mechanistic enhancement, which is not demonstrated here. "Increase the frequency of HGT" may be more accurate.

Thank you for this comment. We agree that the phrase "facilitate HGT" may imply a mechanistic role that is not supported by our data. To avoid overinterpretation, we have revised the text to use the more accurate wording "increase the frequency of HGT," as suggested by the reviewer.

Line 319 – This event appears to have occurred in the past; please adjust verb tense accordingly.

Thank you for this helpful comment. We agree that past tense is more appropriate when referring to historical HGT events.

Accordingly, we have revised the sentence to:

"Notably, most of the putative HTGs were phage-related genes, which suggests that HGT involving *B. glumae* was primarily mediated by phages."

Line 366 – The use of "may" is confusing in this context. If the strain is a clinical isolate, it must have caused infection at some point. Consider rephrasing to something like: "The strain may further increase pathogenicity" or "The isolate shows genomic traits consistent with pathogenic potential."

Thank you for this helpful comment. We agree that the original phrasing could create confusion. The clinical isolation mentioned in our manuscript refers to a strain reported in a previous study, not to the Thai strains analyzed here. To clarify this point and avoid misunderstanding, we have revised the sentence as follow:

The original text:

"Given that *B. glumae* has been isolated from an immunocompromised patient, it may already have the capacity to cause human infections [3]"

Has been revised to:

"A previous report of *B. glumae* isolated from an immunocompromised patient [3] demonstrates that the species has the capacity to cause human infection."

Figures

Figure 1 – The rationale for including all listed strains is unclear, and the naming convention is nonstandard. Please explain the context, significance, and nomenclature choice.

Thank you for this helpful comment. The non-Thai strains included in Figure 1 were selected from publicly available genome databases to represent the global diversity of *B. glumae*. Including these reference strains enables contextual interpretation of the Thai isolates by allowing us to identify whether they cluster with previously described international lineages or form unique Thai-specific clades. This is essential for evaluating hypotheses about multiple introductions versus local expansion. To clarify this rationale, we have revised the text in the Results section where Figure 1 is introduced.

The original text:

"The genomes of the 16 Thai *B. glumae* strains were analyzed alongside those of strains from other countries to investigate their phylogenetic relationships."

Has been revised to:

"The genomes of the 16 Thai *B. glumae* strains were analyzed alongside publicly available genomes from multiple countries to contextualize the evolutionary relationships of Thai strains within the global population structure."

In terms of nomenclature, we retained the original strain names assigned by the submitting laboratories in NCBI to maintain source accuracy, reproducibility, and consistency with prior publications. To improve clarity, we have added an explanation in the Figure 1 legend noting that the strain names follow the original designations recorded in public databases.

The original figure legend:

"A maximum-likelihood tree was constructed using core-genome SNPs from the Thai *B. glumae* isolates and publicly available genomes. Bootstrap support values are indicated by circles at the nodes."

Has been revised to:

"A maximum-likelihood tree was constructed using core-genome SNPs from the Thai *B. glumae* isolates and publicly available genomes. Strain names are shown as originally recorded in public genome databases. Bootstrap support values are indicated by circles at the nodes."

Figure 2 – The figure titles are rotated and difficult to read. Please reformat for clarity and ensure proper orientation.

We appreciate the reviewer's helpful comment regarding this point. We have reformatted Figure 2 to improve readability by correcting the orientation of all titles and labels. The updated figure now presents all text in a standard, readable orientation for clearer interpretation.

Figure 3 – The figure's implications are unclear. Colors are difficult to distinguish, and no numerical percentages are provided. The category "Other" further complicates interpretation. Please explain the biological relevance of the data shown — why the relative percentages matter and how they support your conclusions.

Thank you for this helpful comment. We have revised Figure 3 to improve clarity and interpretability. The following updates have been made:

1. Improved color palette:

We applied a high-contrast palette to ensure that all categories can be easily distinguished.

2. Added numerical labels:

Percentage values are now shown directly on each pie slice to allow quantitative interpretation of the distribution.

3. Clarified the "Other" category:

In panel B (distribution by function), the "Other" category represents functional groups that individually accounted for less than 1% of all predicted horizontally transferred genes. The figure legend has been revised as follows for improved clarity:

"Fig 3. Distribution of the putative horizontally transferred genes present in the B. glumae genomes. (A) Distribution by species group. (B) Distribution by function. Percentage values are indicated within each segment. Functional categories that represent less than 1% of total HTGs are combined under "Other"."

Regarding the biological relevance, Figure 3 summarizes the relative contribution of donor taxa (panel A) and functional categories (panel B) among the HTGs identified across the *B. glumae* genomes. These distributions provide insight into:

1. Likely donor lineages involved in horizontal gene transfer, supporting conclusions about interspecies gene flow.
2. Functional roles of the transferred genes such as phage-related, mobile genetic elements, regulatory, and metabolic functions which help infer the potential biological impact of HGT on genome evolution.

The enrichment of phage-associated functions is consistent with our finding that Thai *B. glumae* isolates harbor multiple prophages and supports the hypothesis that phages play an important role in mediating HTGs in *B. glumae*.

Thus, the relative percentages in Figure 3 help contextualize how HGT may shape genomic plasticity, and they strengthen the conclusion that phage-mediated transfer appears to be a major contributor to the horizontal gene pool in *B. glumae*.
